# Handgrip and leg strength and risk of severe fall injuries in adults aged ≥ 80 years: a prospective HUNT study

**DOI:** 10.1186/s12877-026-08012-6

**Published:** 2026-07-21

**Authors:** Jonathan Berg, Javaid Nauman, Håvard Dalen, Håvard K Skjellegrind, Pernille Thingstad, Øyvind O Salvesen, Stuart M Phillips, Ulrik Wisløff

**Affiliations:** 1 Department of Circulation and Medical Imaging, Cardiac Exercise Research Group, Faculty of Medicine and Health Sciences, Norwegian University of Science and Technology, Postboks 8905, Trondheim, 7491 Norway; 2 Department of Cardiology and Cardiothoracic Surgery, St. Olav’s University Hospital, Trondheim, Norway; 3 Institute of Public Health, College of Medicine and Health Sciences, United Arab Emirates University, Al-Ain, United Arab Emirates; 4 Department of Medicine, Levanger Hospital, Nord-Trøndelag Hospital Trust, Levanger, Norway; 5 HUNT Research Centre, Department of Public Health and Nursing, Faculty of Medicine and Health Sciences, Norwegian University of Science and Technology, Levanger, Norway; 6 Levanger Hospital, Nord-Trøndelag Hospital Trust, Levanger, Norway; 7 Department of Neuromedicine and Movement Science, Faculty of Medicine and Health Sciences, Norwegian University of Science and Technology, Trondheim, Norway; 8 Health and Care Services, The Municipality of Trondheim, Trondheim, Norway; 9 Department of Public Health and Nursing, Faculty of Medicine and Health Sciences, Norwegian University of Science and Technology, Trondheim, Norway; 10 Department of Kinesiology, McMaster University, Hamilton, ON Canada; 11 School of Human Movement & Nutrition Sciences, University of Queensland, St Lucia, QLD Australia

**Keywords:** Ageing, Fall prevention, Functional decline, Muscle strength, Sarcopenia

## Abstract

**Background:**

Adults aged ≥ 80 years experience the highest rates of fall-related injury and mortality, yet remain underrepresented in prospective fall research. Although reduced muscle strength is recognised as a risk factor for falls, prospective evidence linking muscle strength to severe fall injuries in the oldest old remains limited.

**Methods:**

This prospective cohort study included 3059 individuals aged ≥ 80 years from the fourth Trøndelag Health Study (HUNT4) 70 + Survey in Norway. Using sex-specific reference values, handgrip and leg strength were classified as strong, moderately reduced, or reduced. Cox proportional hazards regression was used to examine associations between strength categories and severe fall injuries, ascertained from national health registries.

**Results:**

Baseline median age was 84 years (interquartile range 82–89; 59% women). Over 3.4 years, 223 individuals experienced either a non-fatal (*n* = 196) or fatal (*n* = 27) fall injury. Those with strong versus reduced handgrip and leg strength had a lower risk (adjusted hazard ratios of 0.63 [95% CI 0.43–0.92] and 0.60 [95% CI 0.43–0.86], respectively). In sex-stratified analyses, the association with handgrip strength was evident only in women, whereas strong leg strength was associated with lower risk in both sexes. Specifically, women with handgrip strength ≥ 22.2 kg and men and women with leg strength corresponding to completing the five-times-sit-to-stand test in ≤ 13.2 and ≤ 14.3 s, respectively, had a markedly lower risk of severe fall injuries.

**Conclusions:**

Among very old Norwegian adults, greater muscular strength was associated with a lower risk of severe fall injuries. Our findings provide clinically interpretable strength thresholds that may support fall injury risk assessment and targeted prevention strategies among the oldest old.

**Supplementary Information:**

The online version contains supplementary material available at https://doi.org/10.1186/s12877-026-08012-6.

## Background

With 37.3 million annual falls requiring medical attention, falls constitute a significant global health challenge and are a leading cause of death among older adults [1, 2]. In Norway, falls were identified as the second most costly health condition in 2019, incurring more than 14 billion NOK in direct healthcare expenses [3]. The risk of falls and sustaining serious injuries after falls escalates with advancing age, driven by medical, sensory, and cognitive factors [4, 5]. Fall-related mortality is approximately twenty times higher in men and nine times higher in women aged ≥ 85 years compared to those aged 65–74, demonstrating a disproportionate vulnerability of the oldest cohort [6].

Recognised in the World Guidelines for Falls Prevention as a modifiable risk factor, low muscular strength is an independent predictor of falls among older adults [7–9]. Low muscular strength is a key component of sarcopenia and frailty, and is closely linked to mortality and independence [10]. Strength begins to decline as early as the fourth decade, with a steeper decline after age 80 [11]. Despite having the highest burden of fall-related injury and mortality, older adults ≥ 80 years remain underrepresented in prospective fall research. Furthermore, because advancing age is associated not only with lower muscular strength but also with a higher prevalence of other risk factors, the association between muscular strength and falls may be diminished. Accordingly, a recent cross-sectional study demonstrated that handgrip strength was significantly inversely associated with falls in older adults aged 60–75 years, but not in those aged ≥ 75 [9]. However, like many other studies, this study was cross-sectional and relied on self-reports of falls [9]. Evidence remains limited among adults aged 80 years and older, using standardised upper- and lower-body strength assessments alongside registry-based severe fall-injury outcomes. Therefore, we aimed to examine whether muscular strength was prospectively associated with the risk of severe non-fatal and fatal fall injuries among older adults aged ≥ 80 years, using registry-based outcome data.

## Methods

### Study design and population

**Fig. 1:**
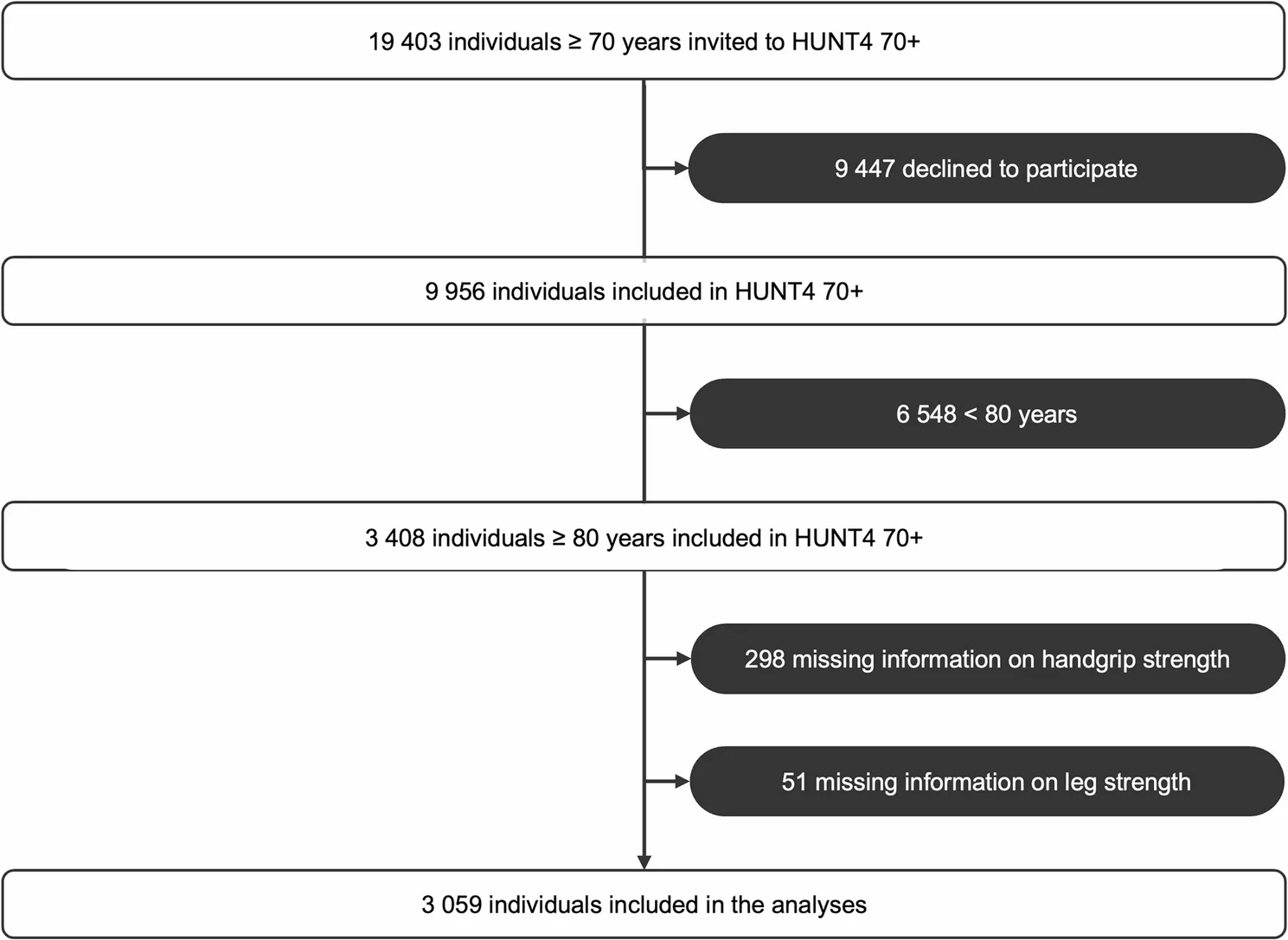
Flowchart of participants

This prospective cohort study included individuals ≥ 80 years from the 70-year-and-older substudy of the fourth wave of the Trøndelag Health Study (HUNT4), called HUNT4 70+ (2017–2019). HUNT4 70 + was designed to facilitate participation among older and frail individuals by offering examinations at field stations, in private homes, and in nursing homes. A recently published cohort profile reported no systematic differences in common chronic diagnoses between HUNT4 70 + participants and invited participants [12]. Further details of data collection are described elsewhere [13, 14]. Our study focused on individuals ≥ 80 years, given their high risk of falls and fall-related injuries, and their underrepresentation in previous research. Of the 3408 individuals ≥ 80 years, we included 3059 with complete handgrip and leg strength data (Fig. 1). Details of the included versus excluded participants are shown in Additional file 1, Table S1.

### Muscular strength

According to the Southampton protocol, handgrip strength was measured using a Jamar digital dynamometer [15]. Following a practice trial, participants completed three maximal trials per hand. We used the peak value from the six trials. Detailed information on the calibration status of individual dynamometers prior to testing was unavailable. However, the Southampton protocol specifies that dynamometers should be calibrated in accordance with manufacturer guidance, which recommends calibration at least annually (or more frequently with heavy use), and standard equipment maintenance in the study would be expected to follow this schedule. Participants without a valid handgrip measurement, including those with a recorded value of 0.0 kg (*n* = 16), were treated as missing because the dynamometer registers force even with minimal effort, making non-performance unlikely to reflect true muscle weakness. Leg strength was estimated using the five-times-sit-to-stand test from the Short Physical Performance Battery [16]. Although commonly used as a proxy for lower-limb strength, the five-times-sit-to-stand test reflects broader lower-limb function and does not isolate muscle strength from other components of physical function [17]. The time elapsed between the first and fifth rises was recorded, with an inability to complete the test defined as taking more than 60 s, using hands for support, or being unable to rise. Participants unable to complete the test were retained in the analyses and classified as having reduced leg strength.

In accordance with the 2019 Framework from the European Working Group on Sarcopenia in Older People (EWGSOP2), participants were classified into three categories (reduced, moderately reduced, and strong) based on sex-specific normative reference values representing peak strength in adulthood from a healthy Norwegian reference population [10, 18]. For handgrip strength, participants were categorised as having moderately reduced or reduced strength if their handgrip strength measurements were 2-2.99 or ≥ 3.0 standard deviations (SDs) below the sex-specific highest observed mean (Additional file 1, Table S2) [18]. For leg strength, based on chair stand time, moderately reduced or reduced strength was defined as measurements 2-2.99 or ≥ 3.0 SDs above the sex-specific lowest observed mean time, respectively (Additional file 1, Table S2) [18]. Participants with handgrip strength within two SDs or above the reference mean, or chair stand time within two SDs of, or faster than the sex-specific reference [18], were classified as ‘strong’. These cut-points were chosen a priori based on the EWGSOP2 framework and external normative reference values and were not derived from the present study.

### Other variables

We calculated body mass index (BMI; kg/m^2^) from height and body mass, measured to the nearest centimetre and decimal, respectively. Balance performance was assessed using a one-legged standing test [19]. The test was performed on a self-selected limb, without assistive devices, and with eyes open for up to 30 s or until the participant could no longer maintain the position [19]. According to the Diagnostic and Statistical Manual of Mental Disorders (DSM-5) [20], two specialists assessed and categorised each participant into no cognitive impairment, mild cognitive impairment, dementia, or other cognitive impairment. More details on the cognitive evaluation are available elsewhere [14]. For analyses, we dichotomised cognitive status as cognitive impairment (yes [mild cognitive impairment, dementia, or other cognitive impairment]) or no [no cognitive impairment]. Questionnaire data included education level (≤ 10 years, 11–13 years, and ≥ 14 years of education based on the highest attained educational degree) and self-reported history of cardiovascular disease (angina pectoris, atrial fibrillation, heart failure, or myocardial infarction).

### Endpoints follow-up

Using participants’ unique personal identification numbers, we linked HUNT4 70 + data to the Norwegian Patient Registry and the Norwegian Cause of Death Registry to identify fatal and non-fatal falls, classified according to the International Classification of Diseases, Tenth Revision (ICD-10) codes W00–19. We defined the primary endpoint as the first occurrence of a non-fatal or fatal fall. Non-fatal falls were defined as all falls requiring hospitalisation, emergency department visits, or specialist outpatient treatment within secondary care, whereas fatal falls were defined as all deaths in which falls were the underlying cause. We followed participants from their examination until the date of the event, death from other causes, or the end of follow-up on August 08, 2022.

### Statistical analyses

Continuous variables are reported as means (SD) and medians (interquartile range [IQR]). Categorical variables are summarised as numbers and percentages (%). Kaplan-Meier curves illustrate the unadjusted event risk across strength categories. We used Cox proportional hazards regression to calculate hazard ratios (HRs) with 95% confidence intervals (CIs) for the prospective association between handgrip and leg strength and our primary composite endpoint. We evaluated the proportional hazards assumption by investigating Schoenfeld residuals. Overall, the evaluation did not indicate a clear violation of the proportional hazards assumption. For leg strength, the exposure term showed borderline evidence of possible non-proportionality, whereas the global tests in the fully adjusted models were not statistically significant. Visual inspection of the scaled Schoenfeld residuals did not suggest a clear systematic time trend. The time scale was the number of days between the examination and censoring dates. All analyses were adjusted for baseline age and sex (Model 1), and further for baseline BMI, self-reported history of cardiovascular disease (yes, no), and cognitive impairment (yes, no) (Model 2). Model 1 aimed to evaluate the clinical utility of muscle strength as a marker of the risk of severe fall injury, whereas Model 2 estimated its independent association beyond other established risk factors. Missing data on covariates were imputed using multiple imputation by chained equations with 20 imputed datasets and 20 iterations. Body mass index was imputed using predictive mean matching, and cognitive status was imputed using multinomial logistic regression. The imputation model included age, sex, cardiovascular disease, handgrip strength category, leg strength category, the binary and combined strength variables used in supplementary analyses, follow-up time, and the event indicator. Follow-up time and event status were included as predictors but were not imputed. Estimates from the imputed datasets were pooled according to Rubin’s rules. We also conducted analyses stratified by sex. To evaluate whether sex modified the associations, we formally tested interactions between sex and handgrip strength and leg strength in Model 2. For clinical relevance, supplementary binary analyses were performed. These examined the association between handgrip or leg strength being below or above 2.0 SDs from the normative mean, respectively. From the binary handgrip and leg strength classifications, we also estimated the absolute difference in event rates.

To assess the robustness of our findings, we conducted five sensitivity analyses. First, we performed complete case analyses, excluding individuals with missing data on potential confounders. Second, we excluded primary outcome events that occurred during the first year of follow-up to minimise reverse causation bias. Third, we further adjusted for baseline one-leg standing test performance to examine whether the associations between muscle strength and severe fall injuries were influenced by balance performance. Fourth, we examined the combined associations of handgrip and leg strength classifications for the primary endpoint. Finally, because residents in long-term care may be underrepresented in national health registries of medically attended falls, we performed a sensitivity analysis excluding those examined in nursing homes. Analyses were done with IBM SPSS Statistics version 30.0 and RStudio version 2024.12.1.

## Results

### Descriptive data

Table 1 shows the baseline characteristics of the 3059 study participants. Median age was 84 years (IQR 82–89), and 1806 (59.0%) were women. Table 1 shows mean handgrip strength and chair-stand times by sex. A quarter (24.6%) of the men and more than a third (34.5%) of the women were unable to complete the five-times-sit-to-stand test within the allocated time or without using their hands (Table 1). According to our sex-specific cut-off scores, 25.7% and 35.7% of the population had reduced or moderately reduced handgrip strength, respectively. For leg strength, 39.8% and 16.3% were classified as reduced or moderately reduced, respectively.

**Table 1:** Baseline characteristics of participants stratified by sex

	Women	Men
Participants, *n* (%)	1806 (59.0)	1253 (41.0)
Assessment location		
Field station, n (%)	1110 (61.5)	956 (76.3)
Home, n (%)	430 (23.8)	176 (14.0)
Long-term care facility, n (%)	266 (14.7)	121 (9.7)
Median age, years (IQR)	85 (82–89)	84 (81–88)
Mean body mass index, kg/m^2^ (SD)	26.7 (4.7)	26.6 (3.8)
Missing, n (%)	152 (8.4)	81 (6.5)
Mean handgrip strength, kg (SD)	20.3 (5.9)	34.1 (8.7)
Mean five-times-sit-to-stand time, s (SD)	15.1 (7.1)	13.5 (6.3)
Unable five-times-sit-to-stand, n (%)	623 (34.5)	308 (24.6)
Median one-leg standing time, s (IQR)	6.0 (3.1–11.5)	11.1 (4.1–14.9)
Unable one-leg standing, n (%)	902 (49.9)	465 (37.1)
Missing, n (%)	96 (5.3)	53 (4.2)
Education		
Compulsory, n (%)	1050 (58.1)	399 (31.8)
Secondary, n (%)	515 (28.5)	576 (46.0)
Tertiary, n (%)	213 (11.8)	260 (20.8)
Missing, n (%)	28 (1.6)	18 (1.4)
Cardiovascular disease, n (%)	445 (24.6)	482 (38.5)
Cognitive status		
Cognitively healthy, n (%)	645 (35.7)	474 (37.8)
Mild cognitive impairment, n (%)	559 (31.0)	441 (35.2)
Dementia, n (%)	565 (31.3)	315 (25.1)
Other cognitive impairment, n (%)	1 (0.1)	0 (0.0)
Insufficient information, n (%)	36 (2.0)	23 (1.8)

*Abbreviations*: *IQR* Interquartile range, *SD* Standard deviation

### Main results

During a mean follow-up of 3.4 years (SD 1.3, 10554 person-years), 223 participants (7.3%) experienced either a non-fatal or fatal fall. The incidence rate for our primary endpoint was 21.1 events per 1000 person-years in the overall population. Table 2 depicts primary outcome events by handgrip and leg strength classification.

**Table 2:** Risk of non-fatal or fatal fall by handgrip and leg strength, estimated using the five-times-sit-to-stand test

	Variable	*N*	Events	Event rate^a^	Model 1		Model 2	
					HR	95% CI	HR	95% CI
Handgrip strength (kg)								
All	Reduced	787	64	31.5	1	Reference	1	Reference
	Moderately reduced	1092	92	23.7	0.93	0.66–1.30	0.95	0.68–1.33
	Strong	1180	67	14.4	0.60	0.41–0.88	0.63	0.43–0.92
Men	Reduced (< 28.4)	298	12	16.3	1	Reference	1	Reference
	Moderately reduced (28.4–37.6)	525	35	18.6	1.50	0.74–3.01	1.57	0.78–3.17
	Strong (≥ 37.7)	430	19	11.1	1.12	0.50–2.52	1.22	0.54–2.76
Women	Reduced (< 16.6)	489	52	40.2	1	Reference	1	Reference
	Moderately reduced (16.6–22.1)	567	57	28.6	0.79	0.53–1.17	0.79	0.55–1.21
	Strong (≥ 22.2)	750	48	16.3	0.49	0.32–0.76	0.49	0.31–0.77
Five-times-sit-to-stand time (s)								
All	Reduced^b^	1375	112	28.7	1	Reference	1	Reference
	Moderately reduced	436	43	26.8	1.12	0.78–1.62	1.15	0.79–1.66
	Strong	1248	68	13.5	0.61	0.44–0.86	0.60	0.43–0.85
Men	Reduced (> 16.1)^b^	499	28	20.5	1	Reference	1	Reference
	Moderately reduced (13.3–16.1)	204	20	27.5	1.48	0.81–2.71	1.63	0.89-3.00
	Strong (≤ 13.2)	550	18	8.1	0.49	0.26–0.93	0.51	0.27–0.99
Women	Reduced (> 17.6)^b^	876	84	33.0	1	Reference	1	Reference
	Moderately reduced (14.4–17.6)	232	23	26.2	0.91	0.57–1.48	0.91	0.56–1.48
	Strong (≤ 14.3)	698	50	17.8	0.67	0.45–0.99	0.64	0.42–0.96

*Abbreviations*: *HR* Hazard ratio

^a^Incidence rate for primary outcome (non-fatal or fatal fall) per 1000 person-years

^b^Participants unable to complete the five-times-sit-to-stand test were classified as having reduced leg strength

Model 1: Adjusted for baseline age and sex (man, woman)

Model 2: Adjusted for baseline age, sex (man, woman), body mass index, cardiovascular disease (yes, no), and cognitive impairment (yes, no)

**Fig. 2:**
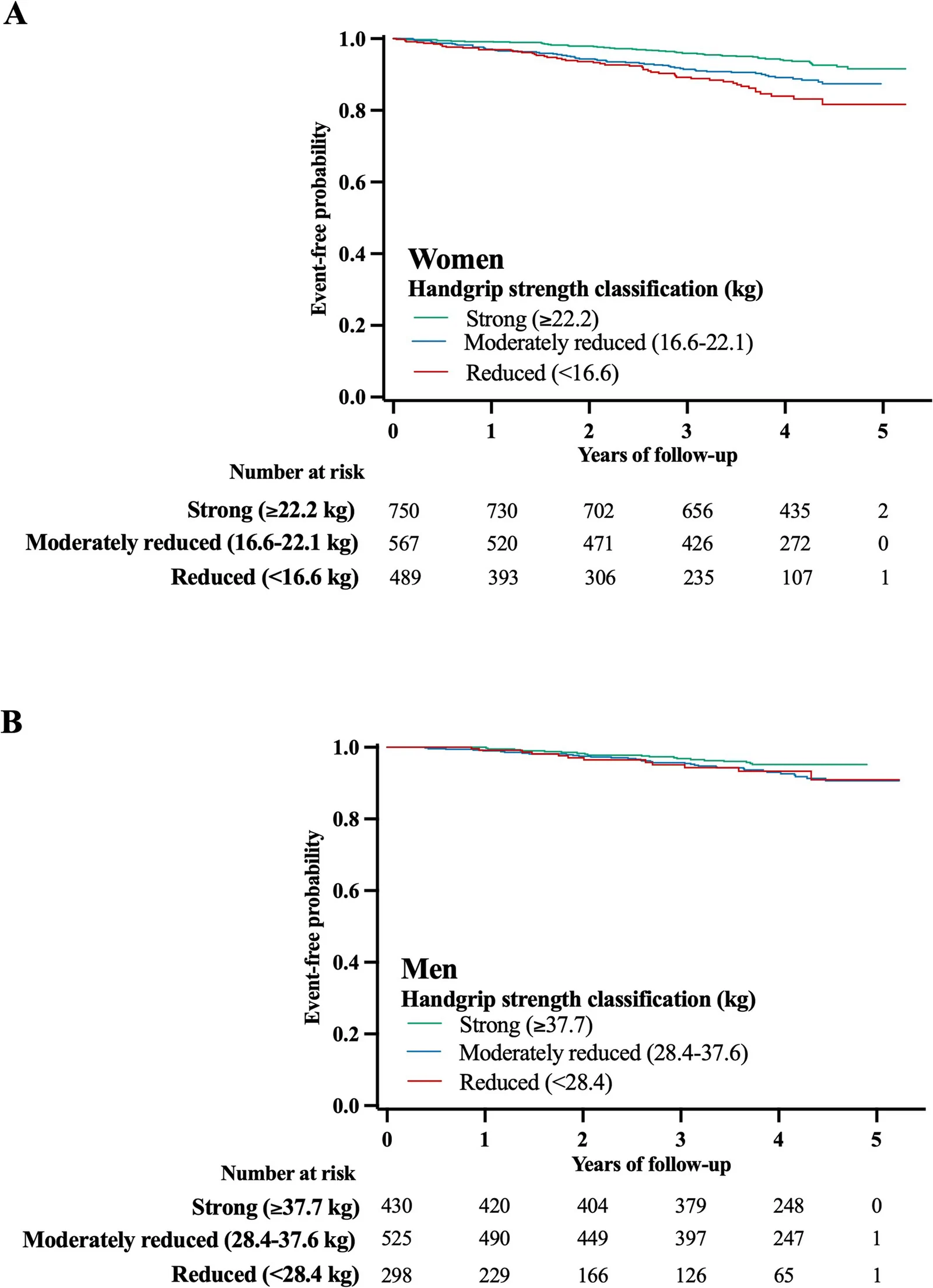
Event-free survival by handgrip strength category. Kaplan-Meier curves showing event-free survival by handgrip strength classification in women (**A**) and men (**B**). Handgrip strength categorised as reduced, moderately reduced, or strong using sex-specific cut-off values. Numbers at risk are shown below each plot

Compared with individuals with reduced handgrip strength, those with strong handgrip strength exhibited a lower risk of the primary endpoint (HR 0.60, 95% CI 0.41–0.88) after adjusting for baseline age and sex (Table 2). Moderately reduced handgrip strength had no predictive value compared with reduced handgrip strength (HR 0.93, 95% CI 0.66–1.30) (Table 2). Adjusting for additional confounders had little impact on our estimates for those with moderately reduced (aHR 0.95, 95% CI 0.68–1.33) or strong handgrip strength (aHR 0.63, 95% CI 0.43–0.92) compared with those with reduced handgrip strength (Table 2). Sex-stratified analyses suggested different patterns for men and women, although there was no statistically significant evidence of an interaction between sex and handgrip strength (*p* = 0.43). For women, HRs in the fully adjusted models were 0.79 (95% CI 0.55–1.21) for those with moderately reduced handgrip strength and 0.49 (95% CI 0.31–0.77) for those with strong handgrip strength versus those with reduced handgrip strength (Table 2; Fig. 2). Compared to men with reduced handgrip strength, HRs were 1.57 (95% CI 0.78–3.17) for men with moderately reduced handgrip strength and 1.22 (95% CI 0.54–2.76) for men with strong handgrip strength in the fully adjusted model (Table 2; Fig. 2).

**Fig. 3:**
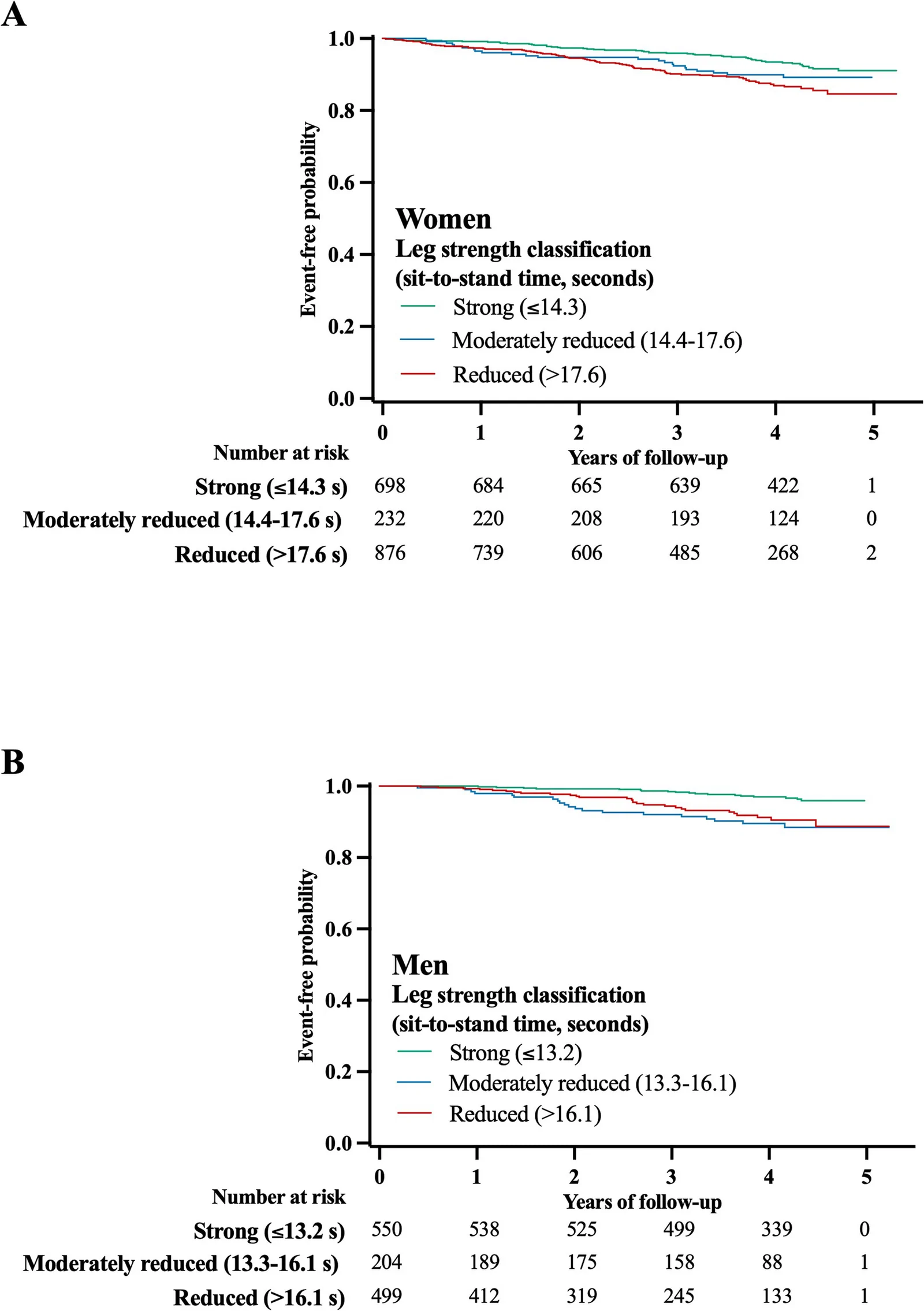
Event-free survival by leg strength category. Kaplan-Meier curves showing event-free survival according to leg strength classification, assessed by sit-to-stand test performance, in women (**A**) and men (**B**). Leg strength categorised as reduced, moderately reduced, or strong using sex-specific cut-off values. Participants unable to complete the five-times-sit-to-stand test were classified as having reduced leg strength. Numbers at risk are shown below each plot

Corresponding estimates for leg strength showed a lower risk for the primary endpoint among those with strong versus reduced leg strength in both minimally adjusted (HR 0.61, 95% CI 0.44–0.86) and fully adjusted models (aHR 0.60, 95% CI 0.43–0.85) (Table 2). HRs for having moderately reduced versus reduced leg strength were 1.12 (95% CI 0.78–1.62) and 1.15 (95% CI 0.79–1.66) in the minimally and fully adjusted models, respectively. When stratified by sex, strong leg strength was associated with a lower risk of the primary endpoint in both women and men, with no evidence of a sex interaction (*p* = 0.11). Compared to women with reduced leg strength, women with moderately reduced leg strength showed no lower risk of the primary endpoint (aHR 0.91, 95% CI 0.56–1.48) (Table 2; Fig. 3). Women with strong leg strength had a 36% lower risk of a non-fatal or fatal fall injury than those with reduced leg strength (aHR 0.64, 95% CI 0.42–0.96) (Table 2; Fig. 3). For men, HRs in the fully adjusted model were 1.63 (95% CI 0.89-3.00) for those with moderately reduced leg strength and 0.51 (95% CI 0.27–0.99) for those with strong leg strength compared to those with reduced leg strength (Table 2; Fig. 3).

Among the 1056 women with handgrip strength more than 2 SDs below the sex-specific normative mean, 109 (10.3%) experienced a non-fatal or fatal fall, compared with 48 (6.4%) of the 750 women with handgrip strength at or above the threshold of 22.2 kg. The absolute difference in event rates was 16.9 events per 1000 person-years, with an adjusted HR of 0.57 (95% CI 0.40–0.83) (Table 3). Of the 703 men with sit-to-stand times exceeding the 2 SD threshold above the normative mean, 48 (6.8%) experienced a non-fatal or fatal fall. In comparison, only 18 (3.3%) men with sit-to-stand times below the threshold of 13.2 s experienced a non-fatal or fatal fall. Among men, the event rate was 22.9 per 1000 person-years in those with five-repetition sit-to-stand times > 13.2 s or unable to complete the test, compared with 8.1 per 1000 person-years in those completing the test in ≤ 13.2 s. This represented a difference of 14.8 fewer events per 1000 person-years. In the fully adjusted model, men completing the test in ≤ 13.2 s had a 59% lower risk of a non-fatal or fatal fall injury than those above this threshold or unable to complete the test (aHR 0.41, 95% CI 0.23–0.75) (Table 3). Among women, completing the five-times sit-to-stand test in 14.3 s was associated with 13.5 fewer events per 1000 person-years (aHR 0.65, 95% CI 0.45–0.96) (Table 3). The binary analyses showed no association between handgrip strength and severe fall injury for men (aHR 0.85, 95% CI 0.48–1.52).

**Table 3:** Risk of non-fatal or fatal fall by sex-specific thresholds of handgrip and leg strength, estimated using the five-times-sit-to-stand test

	Variable	*N*	Events	Event rate^a^	Model 1		Model 2	
					HR	95% CI	HR	95% CI
Handgrip strength (kg)								
Men	< 37.7	823	47	18.0	1.00	Reference	1.00	Reference
	≥ 37.7	430	19	11.1	0.81	0.46–1.45	0.85	0.48–1.52
Women	< 22.2	1056	109	33.2	1.00	Reference	1.00	Reference
	≥ 22.2	750	48	16.3	0.57	0.40–0.83	0.57	0.40–0.83
Five-times-sit-to-stand time (s)								
Men	> 13.2^b^	703	48	22.9	1.00	Reference	1.00	Reference
	≤ 13.2	550	18	8.1	0.41	0.23–0.73	0.41	0.23–0.75
Women	> 14.3^b^	1108	107	31.3	1.00	Reference	1.00	Reference
	≤ 14.3	698	50	17.8	0.69	0.48–0.99	0.65	0.45–0.96

*Abbreviations*: *HR* Hazard ratio

^a^Incidence rate for primary outcome (non-fatal or fatal fall) per 1000 person-years

^b^Participants unable to complete the five-times-sit-to-stand test were included in the above-threshold group

Model 1: Adjusted for baseline age and sex (man, woman)

Model 2: Adjusted for baseline age, sex (man, woman), body mass index, cardiovascular disease (yes, no), and cognitive impairment (yes, no)

### Sensitivity analyses

Excluding individuals with missing data on potential confounders had little effect on our point estimates (Additional file 1, Table S3). When excluding individuals with events of the primary endpoint during the first year of follow-up, the associations between both handgrip and leg strength were weaker, but the overall direction of the estimates remained consistent (Additional file 1, Table S4). Adjusting for baseline balance performance attenuated the associations between leg strength and the primary endpoint (Additional file 1, Table S5). The direction of the estimates remained, but the magnitude was reduced and the associations were no longer statistically significant in the total sample and in the sex-stratified analyses. The attenuation was most pronounced in women. The association between handgrip strength and the primary endpoint in women was only minimally attenuated. In the fully adjusted model for the combined analyses of leg and handgrip strength, only those classified as having both strong handgrip and leg strength showed a lower risk of the primary endpoint (aHR 0.49, 95% CI 0.29–0.83) (Additional file 1, Table S6). The combined analyses also suggested an increased risk of the primary endpoint for individuals with moderately reduced leg strength and reduced handgrip strength compared to those classified as reduced in both measures of muscle strength (aHR 2.16, 95% CI 1.07–4.34) (Additional file 1, Table S6). Excluding individuals living in long-term care facilities did not materially change the results (Additional file 1, Table S7).

## Discussion

In this prospective cohort study of Norwegian adults aged 80 years and older, higher handgrip and leg strength were clinical markers of a lower risk of registry-verified severe fall injuries. Leg strength was associated with a lower risk in both women and men, whereas handgrip strength was associated with a lower risk only in women. In women, handgrip strength of at least 22.2 kg, and in women and men, chair-stand times below 14.3 and 13.2 s, respectively, identified individuals at reduced risk. After additional adjustment for balance performance, the association between handgrip strength and severe fall injuries in women remained, whereas the associations for leg strength were attenuated. Nonetheless, our findings suggest that simple measures of muscular strength remain important clinical markers of vulnerability to severe fall injuries among the oldest old.

Although the proportion of participants (7.3%) experiencing a non-fatal or fatal fall injury during follow-up may seem low for a population aged 80 years and older, the corresponding incidence rate of 21.1 events per 1000 person-years is comparable to that reported in the ASPirin in Reducing Events in the Elderly (ASPREE) trial [21]. Thus, our event rate appears consistent with previous studies using hospital-based fall-injury outcomes and should be interpreted as reflecting severe fall injuries captured in secondary care, rather than all falls and fall injuries among adults aged 80 years and older.

To date, this is the only prospective study to examine the relationship between upper- and lower-body muscle strength and the risk of severe fall injuries in very old adults. The beneficial effect of muscle strength observed here aligns with a 2019 meta-analysis indicating that sarcopenia, the clinical condition characterised by age-related declines in muscle strength and mass, is linked to a 70% increased risk of fractures [22]. Furthermore, consistent with our findings, results from the ASPREE trial demonstrated that individuals with handgrip strength in the first versus fifth quintile had a 73% higher risk of a fall requiring hospital visits during a four-year follow-up period [21]. However, because participants in our study were approximately ten years older than those in ASPREE, our results extend prior evidence by demonstrating that the relationship between muscle strength and fall injury persists into more advanced age [21]. Additionally, our findings demonstrate that both upper- and lower-body muscle strength are indicators of future risk of severe fall injuries. Conversely, a recent cross-sectional study utilising data from the China Health and Retirement Longitudinal Study (CHARLS) found that lower handgrip strength was associated with an increased risk of falls in older adults aged 60 to 75 years, but not in those aged 75 or older [9]. The authors suggested that the predictive power of muscle strength for falls might diminish due to a notable increase in other fall-related risk factors [9]. However, the CHARLS study relied on retrospectively self-reported falls [9]. In contrast, we prospectively documented more severe fall events with the greatest clinical and public health impact. Nevertheless, increased muscle strength may protect against more severe fall injuries, potentially by enhancing protective mechanisms during falls, such as rapid upper-limb reactions, reaching for support, and using the arms to attenuate impact [23]. Furthermore, the outcomes after a fall, including hospitalisation, in older adults are strongly associated with the ability to rise independently from the floor, which is closely related to sit-to-stand performance, our surrogate measure of leg strength in this study [24, 25].

The role of balance should also be considered when interpreting findings on leg strength. Although commonly used as a proxy for lower-limb strength, the sit-to-stand test reflects broader lower-limb function and does not isolate muscle strength from other components of physical function [17]. In our sensitivity analyses, further adjustment for one-leg standing performance attenuated the association between leg strength and severe fall injuries, suggesting that balance-related function may partly explain this association. Similarly, inability to complete the test may reflect reduced strength or balance and coordination impairment, or a combination of these factors [26].

Consistent with previous observations, our findings indicate higher event rates among women than among men [27]. In sex-stratified analyses, the association between handgrip strength and severe fall injuries was apparent in women but not in men. However, because formal interaction testing did not provide evidence that sex modified this association, this apparent difference should be interpreted with caution. One possible explanation for the pattern observed in the stratified analyses is sex-specific differences in the ratio of muscle strength to body mass. Aligning with normative values for handgrip strength, women in our cohort exhibited substantially lower absolute (20.3 vs. 34.1 kg) and relative (0.3 vs. 0.5 kg/kg) handgrip strength [28]. Women with reduced handgrip strength might therefore lack sufficient strength to mitigate impact forces during falls, thereby increasing the risk of fall-related injuries [29, 30].

The supplementary binary analyses provide clinically interpretable thresholds for severe fall injury risk in this population. Notably, thresholds associated with lower risk were close to or above the cohort’s average strength and functional performance and were higher than conventional EWGSOP2 cut-points for low muscle strength. In women, handgrip strength ≥ 22.2 kg was associated with lower risk, compared with a cohort mean of 20.3 kg and the EWGSOP2 threshold of < 16 kg. For five-repetition sit-to-stand performance, completing the test in ≤ 13.2 s in men and ≤ 14.3 s in women was associated with lower risk, compared with cohort means of 13.5 and 15.1 s, respectively, and the EWGSOP2 threshold of > 15 s. These findings suggest that average strength and conventional low-strength thresholds may not fully capture clinically relevant gradients in severe fall injury risk among adults aged 80 years and older. However, these thresholds should be interpreted as candidate clinical markers rather than diagnostic cut-points and require validation in other populations.

This study has several notable strengths. It is the first prospective cohort study to examine both handgrip and leg strength in relation to severe fall injuries among adults aged 80 years and older, using registry-verified outcome data. The use of objective, validated strength measures and linkage to national health registries ensures a reliable assessment of exposure and outcomes. Importantly, we were able to follow a relatively large sample of the oldest old, a group often underrepresented in fall-related research.

Several limitations should be acknowledged. Selection bias should be considered when interpreting our findings. Although the 51.3% participation rate is relatively high for an older population-based cohort, and HUNT 4 70 + included procedures to facilitate participation among frail older adults, participation was incomplete, and the present analytic sample required valid handgrip and sit-to-stand data. Individuals excluded from the present analyses were older, more often examined in long-term care facilities, and more often had dementia. Thus, selection bias may have occurred both at participation in HUNT4 70 + and at inclusion in the present analytic sample. Our results may therefore be less generalisable to the frailest adults aged 80 years and older, particularly those living in long-term care. The observational study design limits causal conclusions, although the prospective design and the use of incident registry-based endpoints strengthen temporal interpretation compared with cross-sectional studies or those relying on retrospective fall data. The sit-to-stand test is a well-validated proxy for leg strength, but it does not isolate muscle strength from other components of physical function [17]. Muscle strength was also measured only at baseline, so we could not account for changes in strength during follow-up. However, a single standardised assessment is feasible in clinical practice and may still be useful for identifying individuals at increased risk. Information bias in outcome ascertainment should also be considered. Although registry-based ascertainment reduces recall bias and captures clinically important events, our endpoint did not include falls that were untreated, treated only in primary care, or that did not result in injury. Thus, our findings should be interpreted as applying to severe fall injuries rather than to general fall risk. Lastly, we could not classify muscle strength relative to body mass or BMI because the external reference values used to define our categories were based on absolute strength. We therefore present strength in absolute units, which are clinically interpretable and commonly used in practice [31].

## Conclusions

Our findings emphasise muscular strength as a clinical marker of vulnerability to severe fall injuries in advanced age. We provide clinically interpretable thresholds for handgrip and leg strength that may help clinicians identify high-risk individuals and develop targeted prevention strategies. Future research in diverse populations is necessary to verify the applicability of these thresholds and to determine whether strength-based interventions can reduce severe fall injuries among the oldest old.

## Supplementary Information

Additional file 1: Tables S1-S7. Table S1: Comparison of included and excluded individuals. Table S2: Sex-specific reference values and cut-off thresholds used to categorise handgrip and leg strength. Table S3: Complete case analysis of non-fatal or fatal falls by handgrip and leg strength classifications. Table S4: Risk of non-fatal or fatal fall by handgrip and leg strength classifications with events during first year of follow-up excluded. Table S5: Risk of non-fatal or fatal fall by handgrip and leg strength classifications with additional adjustment for balance performance. Table S6: Risk of non-fatal or fatal fall by combined handgrip and leg strength classifications. Table S7: Risk of non-fatal or fatal fall by handgrip and leg strength classifications with individuals living in long-term care excluded.

## Data Availability

The data used in this study are from participants in The Nord-Trøndelag Health Study (HUNT). Researchers can access the data following approval of applications to the Regional Committees for Medical Health Research Ethics, HUNT, and the Norwegian Institute of Public Health (NIPH).

## Abbreviations

aHR: Adjusted hazard ratio
ASPREE: ASPirin in Reducing Events in the Elderly Trial
BMI: Body mass index
CHARLS: China Health and Retirement Longitudinal Study
CI: Confidence interval
DSM-5: Diagnostic and Statistical Manual of Mental Disorders
EWGSOP2: 2019 Framework from the European Working Group on Sarcopenia in Older People
HR: Hazard ratio
HUNT4: The fourth wave of the Trøndelag Health Study
ICD-10: International Classification of Diseases, Tenth Revision (ICD-10)
IQR: Interquartile range
SD: Standard deviation
