# Supplementary material for: Handgrip and leg strength and risk of severe fall injuries in adults aged ≥ 80 years: a prospective HUNT study
Source: BMC Geriatr. 2026 Jul 21;26:1303. doi: 10.1186/s12877-026-08012-6 (PMC13673740; doi:10.1186/s12877-026-08012-6)
Supplement: Supplementary file 1 [file 12877_2026_8012_MOESM1_ESM.docx]

**Additional file 1**

Handgrip and Leg Strength and Risk of Severe Fall Injuries in Adults Aged ≥80 Years: A Prospective HUNT Study

**Table of Contents**

[Table S1. Comparison of included and excluded individuals	2](#_Toc233188111)

[Table S2. Sex-specific reference values and cut-off thresholds used to categorise handgrip and leg strength	3](#_Toc233188112)

[Table S3. Complete case analysis of non-fatal or fatal falls by handgrip and leg strength classifications	4](#_Toc233188113)

[Table S4. Risk of non-fatal or fatal fall by handgrip and leg strength classifications with events during first year of follow-up excluded.	5](#_Toc233188114)

[Table S5. Risk of non-fatal or fatal fall by handgrip and leg strength classifications with additional adjustment for balance performance.	6](#_Toc233188115)

[Table S6. Risk of non-fatal or fatal fall by combined grip and leg strength classifications.	7](#_Toc233188116)

[Table S7. Risk of non-fatal or fatal fall by grip and leg strength classifications with individuals living in long-term care excluded.	8](#_Toc233188117)

| Table S1. Comparison of included and excluded individuals | | | |
| --- | --- | --- | --- |
|  | **Included** |  | **Excluded** |
| Participants, n | 3059 |  | 349 |
| Assessment location  Field station, n (%)  Home, n (%)  Long-term care facility, n (%) | 2066 (67.5)  606 (19.8)  387 (12.7) |  | 179 (51.3)  43 (12.3)  127 (36.4) |
| Female sex, n (%) | 1806 (59.0) |  | 220 (63.0) |
| Median age, years (IQR) | 84 (82-89) |  | 86 (82-90) |
| Mean body mass index, kg/m^2^ (SD) | 26.7 (4.4) |  | 26.0 (4.6) |
| Missing, n (%) | 233 (7.6) |  | 139 (39.8) |
| Education |  |  |  |
| Compulsory, n (%) | 1110 (36.3) |  | 177 (50.7) |
| Secondary, n (%) | 996 (32.6) |  | 121 (34.7) |
| Tertiary, n (%) | 445 (14.5) |  | 40 (11.5) |
| Missing, n (%) | 508 (16.6) |  | 11 (3.2) |
| Cardiovascular disease, n (%) | 927 (30.3) |  | 90 (25.8) |
| Cognitive status |  |  |  |
| Cognitively healthy, n (%) | 1119 (36.6) |  | 70 (20.1) |
| Mild cognitive impairment, n (%) | 1000 (32.7) |  | 70 (20.1) |
| Dementia, n (%) | 880 (28.8) |  | 155 (44.4) |
| Other cognitive impairment, n (%) | 1 (0.0) |  | 0 (0.0) |
| Insufficient information, n (%) | 59 (1.9) |  | 54 (15.5) |

| Table S2. Sex-specific reference values and cut-off thresholds used to categorise handgrip and leg strength | | | | | |
| --- | --- | --- | --- | --- | --- |
|  | **Handgrip strength** | | | | |
| **Sex** | **Reference mean (kg)** | **Reference SD (kg)** | **Reduced** | **Moderately reduced** | **Strong** |
| **Men** | 56.4 | 9.4 | <28.4 | 28.4-37.6 | ≥37.7 |
| **Women** | 33.4 | 5.6 | <16.6 | 16.6-22.1 | ≥22.2 |
|  | **Leg strength (sit-to-stand)** | | | | |
|  | **Reference mean (s)** | **Reference SD (s)** | **Reduced** | **Moderately reduced** | **Strong** |
| **Men** | 7.5 | 2.9 | >16.1 | 13.3-16.1 | ≤13.2 |
| **Women** | 7.8 | 3.3 | >17.6 | 14.4-17.6 | ≤14.3 |

SD, standard deviation

Reference values represent sex-specific peak adult performance from the external Norwegian reference population, defined as the highest observed mean handgrip strength and the lowest observed mean time for five-repetition sit-to-stand performance. In accordance with the 2019 framework from the European Working Group on Sarcopenia in Older People (EWGSOP2), moderately reduced and reduced handgrip strength were defined as 2–2.99 SD and ≥3.0 SD below the sex-specific peak mean, respectively. For five-repetition sit-to-stand performance, moderately reduced and reduced categories were defined as 2–2.99 SD and ≥3.0 SD slower than the sex-specific peak mean time, respectively. These values are deviations from peak adult performance.

| Table S3. Complete case analysis of non-fatal or fatal falls by handgrip and leg strength classifications | | | | | |
| --- | --- | --- | --- | --- | --- |
|  | **Variable** | **Model 1** | | **Model 2** | |
|  |  | **HR** | **95% CI** | **HR** | **95% CI** |
|  | **Handgrip strength (kg)** |  | ***N*** **= 3059** |  | ***N*** **= 2777** |
| **All** | Reduced | 1.00 | Reference | 1.00 | Reference |
|  | Moderately reduced | 0.93 | 0.66-1.30 | 0.88 | 0.62-1.26 |
|  | Strong | 0.60 | 0.41-0.88 | 0.59 | 0.40-0.88 |
|  |  |  | ***N*** **= 1253** |  | ***N*** **= 1154** |
| **Men** | Reduced (<28.4) | 1.00 | Reference | 1.00 | Reference |
|  | Moderately reduced (28.4-37.6) | 1.50 | 0.75-2.97 | 1.46 | 0.72-2.95 |
|  | Strong (≥37.7) | 1.12 | 0.50-2.48 | 1.08 | 0.48-2.45 |
|  |  |  | ***N*** **= 1806** |  | ***N*** **= 1623** |
| **Women** | Reduced (<16.6) | 1.00 | Reference | 1.00 | Reference |
|  | Moderately reduced (16.6-22.1) | 0.79 | 0.53-1.17 | 0.72 | 0.47-1.09 |
|  | Strong (≥22.2) | 0.49 | 0.32-0.76 | 0.47 | 0.30-0.74 |
|  | **Leg strength** |  | ***N*** **= 3059** |  | ***N*** **= 2777** |
| **All** | Reduced^a^ | 1.00 | Reference | 1.00 | Reference |
|  | Moderately reduced | 1.12 | 0.78-1.62 | 1.09 | 0.75-1.59 |
|  | Strong | 0.61 | 0.44-0.86 | 0.59 | 0.41-0.84 |
|  |  |  | ***N*** **= 1253** |  | ***N*** **= 1154** |
| **Men** | Reduced (>16.1)^a^ | 1.00 | Reference | 1.00 | Reference |
|  | Moderately reduced (13.3-16.1) | 1.48 | 0.82-2.68 | 1.51 | 0.82-2.78 |
|  | Strong (≤13.2) | 0.49 | 0.26-0.92 | 0.49 | 0.26-0.94 |
|  |  |  | ***N*** **= 1806** |  | ***N*** **= 1623** |
| **Women** | Reduced (>17.6)^a^ | 1.00 | Reference | 1.00 | Reference |
|  | Moderately reduced (14.4-17.6) | 0.91 | 0.57-1.47 | 0.88 | 0.54-1.45 |
|  | Strong (≤14.3) | 0.67 | 0.45-0.99 | 0.62 | 0.41-0.95 |

HR, hazard ratio

^a^Participants unable to complete the five-times-sit-to-stand test were classified as having reduced leg strength

Model 1: Adjusted for baseline age and sex (man, woman)

Model 2: Adjusted for baseline age, sex (man, woman), body mass index, cardiovascular disease (yes, no), and cognitive impairment (yes, no).

| Table S4. Risk of non-fatal or fatal fall by handgrip and leg strength classifications with events during first year of follow-up excluded. | | | | | | | | |
| --- | --- | --- | --- | --- | --- | --- | --- | --- |
|  | **Variable** | ***N*** | **Events** | **Event rate^a^** | **Model 1** | | **Model 2** | |
|  |  |  |  |  | **HR** | **95% CI** | **HR** | **95% CI** |
|  | **Handgrip strength** | |  |  |  |  |  |  |
| **All** | Reduced | 622 | 48 | 24.7 | 1.00 | Reference | 1.00 | Reference |
|  | Moderately reduced | 1010 | 71 | 18.6 | 0.89 | 0.61-1.32 | 0.91 | 0.62-1.34 |
|  | Strong | 1150 | 61 | 13.2 | 0.70 | 0.46-1.06 | 0.72 | 0.47-1.10 |
| **Men** | Reduced (<28.4) | 229 | 10 | 14.3 | 1.00 | Reference | 1.00 | Reference |
|  | Moderately reduced (28.4-37.6) | 490 | 30 | 16.1 | 1.50 | 0.70-3.22 | 1.58 | 0.74-3.39 |
|  | Strong (≥37.7) | 420 | 19 | 11.2 | 1.32 | 0.56-3.13 | 1.45 | 0.61-3.43 |
| **Women** | Reduced (<16.6) | 393 | 38 | 30.6 | 1.00 | Reference | 1.00 | Reference |
|  | Moderately reduced (16.6-22.1) | 520 | 41 | 20.8 | 0.73 | 0.46-1.16 | 0.71 | 0.45-1.14 |
|  | Strong (≥22.2) | 730 | 42 | 14.3 | 0.55 | 0.34-0.90 | 0.53 | 0.32-0.88 |
|  | **Leg strength** |  |  |  |  |  |  |  |
| **All** | Reduced^b^ | 1151 | 87 | 23.0 | 1.00 | Reference | 1.00 | Reference |
|  | Moderately reduced | 409 | 31 | 19.5 | 0.98 | 0.64-1.50 | 1.00 | 0.65-1.53 |
|  | Strong | 1222 | 62 | 12.3 | 0.68 | 0.47-0.97 | 0.67 | 0.46-0.97 |
| **Men** | Reduced^b^ (>16.1) | 412 | 25 | 19.0 | 1.00 | Reference | 1.00 | Reference |
|  | Moderately reduced (13.3-16.1) | 189 | 16 | 22.3 | 1.29 | 0.67-2.48 | 1.42 | 0.73-2.76 |
|  | Strong (≤13.2) | 538 | 18 | 8.1 | 0.52 | 0.27-1.02 | 0.55 | 0.28-1.09 |
| **Women** | Reduced^b^ (>17.6) | 739 | 62 | 25.1 | 1.00 | Reference | 1.00 | Reference |
|  | Moderately reduced (14.4-17.6) | 220 | 15 | 17.3 | 0.77 | 0.43-1.38 | 0.77 | 0.43-1.38 |
|  | Strong (≤14.3) | 684 | 44 | 15.7 | 0.76 | 0.49-1.17 | 0.72 | 0.46-1.13 |
| HR, hazard ratio  ^a^Incidence rate for primary outcome (non-fatal or fatal fall) per 1000 person-years  ^b^Participants unable to complete the five-times-sit-to-stand test were classified as having reduced leg strength  Model 1: Adjusted for baseline age and sex (man, woman)  Model 2: Adjusted for baseline age, sex (man, woman), body mass index, cardiovascular disease (yes, no), and cognitive impairment (yes, no). | | | | | | | | |

| Table S5. Risk of non-fatal or fatal fall by handgrip and leg strength classifications with additional adjustment for balance performance. | | | | | | | | |
| --- | --- | --- | --- | --- | --- | --- | --- | --- |
|  | **Variable** | ***N*** | **Events** | **Event rate^a^** | **Model 2^b^** | | **Model 2^c^** | |
|  |  |  |  |  | **HR** | **95% CI** | **HR** | **95% CI** |
|  | **Handgrip strength** | |  |  |  |  |  |  |
| **All** | Reduced | 732 | 59 | 30.9 | 1.00 | Reference | 1.00 | Reference |
|  | Moderately reduced | 1041 | 89 | 24.0 | 0.96 | 0.67-1.36 | 1.00 | 0.71-1.42 |
|  | Strong | 1137 | 63 | 14.0 | 0.60 | 0.40-0.90 | 0.67 | 0.45-1.00 |
| **Men** | Reduced (<28.4) | 282 | 12 | 17.0 | 1.00 | Reference | 1.00 | Reference |
|  | Moderately reduced (28.4-37.6) | 499 | 33 | 18.4 | 1.56 | 0.77-3.17 | 1.67 | 0.82-3.40 |
|  | Strong (≥37.7) | 419 | 18 | 10.8 | 1.19 | 0.52-2.73 | 1.30 | 0.57-2.99 |
| **Women** | Reduced (<16.6) | 450 | 47 | 39.2 | 1.00 | Reference | 1.00 | Reference |
|  | Moderately reduced (16.6-22.1) | 542 | 56 | 29.3 | 0.80 | 0.53-1.21 | 0.82 | 0.55-1.24 |
|  | Strong (≥22.2) | 718 | 45 | 15.9 | 0.47 | 0.29-0.74 | 0.53 | 0.33-0.85 |
|  | **Leg strength** |  |  |  |  |  |  |  |
| **All** | Reduced^d^ | 1268 | 101 | 27.9 | 1.00 | Reference | 1.00 | Reference |
|  | Moderately reduced | 422 | 43 | 27.6 | 1.20 | 0.83-1.75 | 1.30 | 0.89-1.90 |
|  | Strong | 1220 | 67 | 13.6 | 0.62 | 0.43-0.88 | 0.73 | 0.51-1.05 |
| **Men** | Reduced^d^ (>16.1) | 463 | 26 | 20.4 | 1.00 | Reference | 1.00 | Reference |
|  | Moderately reduced (13.3-16.1) | 202 | 20 | 27.7 | 1.67 | 0.90-3.12 | 1.80 | 0.95-3.41 |
|  | Strong (≤13.2) | 535 | 17 | 7.8 | 0.52 | 0.26-1.02 | 0.57 | 0.28-1.17 |
| **Women** | Reduced^d^ (>17.6) | 805 | 75 | 32.0 | 1.00 | Reference | 1.00 | Reference |
|  | Moderately reduced (14.4-17.6) | 220 | 23 | 27.6 | 0.97 | 0.59-1.58 | 1.04 | 0.64-1.70 |
|  | Strong (≤14.3) | 685 | 50 | 18.1 | 0.65 | 0.43-0.98 | 0.79 | 0.52-1.21 |
| HR, hazard ratio  ^a^Incidence rate for primary outcome (non-fatal or fatal fall) per 1000 person-years  ^b^Model 2 restricted to participants with available one-leg standing test data. Adjusted for baseline age, sex (man, woman), body mass index, cardiovascular disease (yes, no), and cognitive impairment (yes, no).  ^c^Model 2 restricted to participants with available one-leg standing test data. Additionally adjusted for one-leg standing time (seconds).  ^d^Participants unable to complete the five-times-sit-to-stand test were classified as having reduced leg strength | | | | | | | | |

| Table S6. Risk of non-fatal or fatal fall by combined grip and leg strength classifications. | | | | | | | | |
| --- | --- | --- | --- | --- | --- | --- | --- | --- |
| **Variable** |  | ***N*** | **Events** | **Event rate^a^** | **Model 1** | | **Model 2** | |
|  |  |  |  |  | **HR** | **95% CI** | **HR** | **95% CI** |
| **Grip strength** | **Leg strength** |  |  |  |  |  |  |  |
| Reduced^b^ | Reduced^b^ | 625 | 43 | 29.4 | 1.00 | Reference | 1.00 | Reference |
| Moderately reduced | Reduced^b^ | 470 | 42 | 28.9 | 1.10 | 0.71-1.70 | 1.12 | 0.73-1.74 |
| Strong | Reduced^b^ | 280 | 27 | 27.2 | 1.09 | 0.66-1.80 | 1.13 | 0.68-1.88 |
| Reduced^b^ | Moderately reduced | 58 | 10 | 55.1 | 2.09 | 1.04-4.20 | 2.16 | 1.07-4.34 |
| Moderately reduced | Moderately reduced | 179 | 23 | 35.4 | 1.51 | 0.89-2.56 | 1.57 | 0.92-2.68 |
| Strong | Moderately reduced | 199 | 10 | 12.9 | 0.59 | 0.29-1.20 | 0.61 | 0.29-1.25 |
| Reduced^b^ | Strong | 104 | 11 | 28.6 | 1.20 | 0.61-2.36 | 1.15 | 0.58-2.29 |
| Moderately reduced | Strong | 443 | 27 | 15.2 | 0.72 | 0.43-1.21 | 0.71 | 0.42-1.20 |
| Strong | Strong | 701 | 30 | 10.4 | 0.48 | 0.29-0.81 | 0.49 | 0.29-0.83 |
| HR, hazard ratio  ^a^Incidence rate for primary outcome (non-fatal or fatal fall) per 1000 person-years  ^b^Participants unable to complete the five-times-sit-to-stand test were classified as having reduced leg strength  Model 1: Adjusted for baseline age and sex (man, woman)  Model 2: Adjusted for baseline age, sex (man, woman), body mass index, cardiovascular disease (yes, no), and cognitive impairment (yes, no). | | | | | | | | |

| Table S7. Risk of non-fatal or fatal fall by grip and leg strength classifications with individuals living in long-term care excluded. | | | | | | | | |
| --- | --- | --- | --- | --- | --- | --- | --- | --- |
|  | **Variable** | ***N*** | **Events** | **Event rate^a^** | **Model 1** | | **Model 2** | |
|  |  |  |  |  | **HR** | **95% CI** | **HR** | **95% CI** |
|  | **Grip strength** |  |  |  |  |  |  |  |
| **All** | Reduced | 504 | 50 | 33.8 | 1.00 | Reference | 1.00 | Reference |
|  | Moderately reduced | 1015 | 85 | 22.9 | 0.83 | 0.57-1.19 | 0.84 | 0.58-1.21 |
|  | Strong | 1153 | 66 | 14.4 | 0.61 | 0.40-0.91 | 0.63 | 0.42-0.94 |
| **Men** | Reduced (<28.4) | 207 | 12 | 20.8 | 1.00 | Reference | 1.00 | Reference |
|  | Moderately reduced (28.4-37.6) | 502 | 33 | 18.0 | 1.14 | 0.57-2.30 | 1.17 | 0.58-2.36 |
|  | Strong (≥37.7) | 423 | 18 | 10.6 | 0.85 | 0.38-1.93 | 0.90 | 0.40-2.04 |
| **Women** | Reduced (<16.6) | 297 | 38 | 42.1 | 1.00 | Reference | 1.00 | Reference |
|  | Moderately reduced (16.6-22.1) | 513 | 52 | 27.8 | 0.75 | 0.49-1.16 | 0.75 | 0.48-1.16 |
|  | Strong (≥22.2) | 730 | 48 | 16.6 | 0.51 | 0.32-0.82 | 0.50 | 0.31-0.81 |
|  | **Leg strength** |  |  |  |  |  |  |  |
| **All** | Reduced^b^ | 1013 | 92 | 28.8 | 1.00 | Reference | 1.00 | Reference |
|  | Moderately reduced | 417 | 42 | 27.2 | 1.13 | 0.77-1.65 | 1.14 | 0.78-1.66 |
|  | Strong | 1242 | 67 | 13.3 | 0.61 | 0.43-0.86 | 0.60 | 0.42-0.85 |
| **Men** | Reduced^b^ (>16.1) | 386 | 25 | 21.5 | 1.00 | Reference | 1.00 | Reference |
|  | Moderately reduced (13.3-16.1) | 198 | 20 | 28.2 | 1.51 | 0.82-2.80 | 1.65 | 0.89-3.08 |
|  | Strong (≤13.2) | 548 | 18 | 8.1 | 0.48 | 0.25-0.93 | 0.51 | 0.26-0.99 |
| **Women** | Reduced^b^ (>17.6) | 627 | 67 | 33.1 | 1.00 | Reference | 1.00 | Reference |
|  | Moderately reduced (14.4-17.6) | 219 | 22 | 26.3 | 0.95 | 0.58-1.57 | 0.94 | 0.57-1.56 |
|  | Strong (≤14.3) | 694 | 49 | 17.5 | 0.69 | 0.46-1.04 | 0.65 | 0.43-0.99 |
| HR, hazard ratio  ^a^Incidence rate for primary outcome (non-fatal or fatal fall) per 1000 person-years  ^b^Participants unable to complete the five-times-sit-to-stand test were classified as having reduced leg strength  Model 1: Adjusted for baseline age and sex (man, woman)  Model 2: Adjusted for baseline age, sex (man, woman), body mass index, cardiovascular disease (yes, no), and cognitive impairment (yes, no). | | | | | | | | |
